# The dietary inflammatory index and its association with the prevalence of hypertension: A cross-sectional study

**DOI:** 10.3389/fimmu.2022.1097228

**Published:** 2023-01-18

**Authors:** Nan Zhou, Zhi-Ping Xie, Qing Liu, Yun Xu, Si-Cheng Dai, Juan Lu, Jia-Yi Weng, Li-Da Wu

**Affiliations:** ^1^ Health Examination Center, Huadong Sanatorium, Wuxi, China; ^2^ Department of Anesthesiology, Huadong Sanatorium, Wuxi, China; ^3^ Department of Cardiology, The Affiliated Suzhou Hospital of Nanjing Medical University, Suzhou Municipal Hospital, Gusu School, Nanjing Medical University, Suzhou, China; ^4^ Department of Cardiology, Nanjing First Hospital, Nanjing Medical University, Nanjing, China

**Keywords:** dietary inflammatory index, NHANES, inflammation, LASSO, hypertension

## Abstract

**Aims:**

We aim to investigate the association of the Dietary Inflammatory Index (DII) with the prevalence of hypertension in a large multiracial population in the United States.

**Methods:**

Participants from the National Health and Nutrition Examination Survey (NHANES) (1999–2018) were included in this cross-sectional study. Dietary information was obtained and used to calculate DII. Blood pressures of participants were measured by experienced examiners. The NHANES used the method of “stratified multistage probability sampling,” and this study is a weight analysis following the NHANES analytic guidance. Weight logistic regression analysis was adopted to investigate the association of hypertension with DII. Least Absolute Shrinkage and Selection Operator (LASSO) regression was carried out to screen the most important dietary factors associated with the risk of hypertension. Moreover, a nomogram model based on key dietary factors was established; the receiver operating characteristic (ROC) curve was used to evaluate the diagnostic power of the nomogram model for screening hypertension risk.

**Results:**

A total of 45,023 participants were included in this study, representing 191 million residents in the United States. Participants with hypertension had an elevated DII compared with those without hypertension. Weight logistic regression showed that an increment of DII was strongly associated with hypertension after adjusting for confounding factors. The nomogram model, based on key dietary factors screened by LASSO regression, showed a favorable discriminatory power with an area under the curve (AUC) of 78.5% (95% CI: 78.5%–79.3%). Results of the sensitivity analysis excluding participants who received any drug treatment were consistent with those in the main analysis.

**Conclusion:**

An increment of DII is associated with the risk of hypertension. For better prevention and treatment of hypertension, more attention should be paid to controlling dietary inflammation.

## Introduction

Hypertension is a very common clinical syndrome characterized by elevated arterial blood pressure (1). The World Health Organization (WHO) estimated that one in five adults has hypertension worldwide; in other words, there are more than 1.3 billion patients with hypertension at present (2). The incidence of hypertension is still on the rise, and the age of onset of hypertension tends to be younger in recent years. Hypertension accounts for the largest number of deaths worldwide and is particularly harmful to the blood vessel, heart, kidney, and brain. Hypertension is closely associated with unhealthy dietary habits, such as heavy alcohol consumption, excessive intake of sodium salt, and low potassium diet (3). A better understanding of the relationship between the risk of hypertension and dietary habits is essential to prevent the onset and development of hypertension.

Inflammation plays a pivotal role in hypertension, as a large number of studies have already demonstrated that systemic inflammation levels of patients with hypertension are elevated, which are closely associated with arterial stiffness (4). Investigators also found that inhibition of inflammation can alleviate hypertension in animal models (5, 6). Moreover, in a recent retrospective study, researchers revealed that assessing systemic inflammation helped in predicting the risk of hypertension (7). Actually, dietary patterns have a great impact on systemic inflammation; for example, Western dietary patterns characterized by a high caloric content and high fat can lead to significantly elevated systemic inflammation (8). On the contrary, the Mediterranean diet, a commonly recognized healthy dietary pattern, was found to have anti-inflammation effects (9). Therefore, it is necessary to establish a comprehensive measurement method to evaluate the dietary inflammatory potential.

The Dietary Inflammatory Index (DII) is a literature-derived index to evaluate the dietary inflammatory potential, firstly proposed by Shivappa et al. (10) in 2014. DII has been validated that it can reflect the levels of systemic inflammation and closely associated with the expressions of blood inflammatory markers, including C-reactive protein (CRP), tumor necrosis factor (TNF)-α, interleukin (IL)-1β, IL-6, and IL-10 (11, 12). At present, DII has already been widely used to explore the roles of diet-induced inflammation in the occurrence and development of various diseases (13–15). Actually, our team previously conducted a cross-sectional study based on the NHANES to explore the association between DII and the risk of coronary heart disease (CHD). We found that an increment of DII closely associated with the incidence of CHD, and the association between DII and CHD was in a nonlinear relationship (16). However, the relationship between hypertension, another chronic cardiovascular disease closely associated with inflammation, and DII remains unclear. Therefore, we conducted this study based on a large multiracial population from the NHANES to further explore the association between hypertension and DII.

## Methods

### Study population

The NHANES is a research program that assesses the health and nutrition status of residents in the United States. It is a continuous cross-sectional survey conducted once every 2 years by the National Center for Health Statistics in the Centers for Disease Control and Prevention. The method of “stratified multistage probability sampling” was used in the NHANES to ensure that the samples were representative. The official website of the NHANES database describes the detailed methods (http://www.cdc.gov/nchs/nhanes.htm, accessed on 11 November 2022) (17). In the present study, 10 consecutive circles of the NHANES that ranged from 1999/2000 to 2017/2018 were included. The exclusion criteria were as follows: 1) age <18 or ≥80 years, 2) estimated glomerular filtration rate (eGFR) <60 ml/min/1.73 m^2^, 3) pregnant individuals, 4) participants without dietary information.

### Dietary information

Dietary information was collected in the mobile examination center (MEC) through a 24-h recall interview by the nutrition methodology working group of the NHANES. As previously published studies did, the DII that included 28 dietary components was calculated according to the protocol provided by Shivappa et al. (10, 16, 18, 19). Briefly, six important systemic inflammation markers (IL-1β, IL-6, IL-4, IL-10, TNF-α, and CRP) were adopted to reflect the inflammation level. In this study, “+1” was assigned if a dietary component increases the levels of CRP, TNF-α, IL-1β, and IL-6 or reduces the levels of IL-4 and IL-10; if a dietary component decreases the levels of CRP, TNF-α, IL-1β, and IL-6 or increases the levels of IL-4 and IL-10, “−1” was assigned. For each individual food parameter, this score was multiplied by the respective food parameter effect score derived from the literature review. All of the food parameter-specific DII scores were then summed to create the overall DII score for each participant in the study, DII = b1 * n1 + b2 * n2……….b28 * n28, where b refers to the literature-derived inflammatory effect score for each of the evaluable food parameters and n refers to the food parameter-specific percentiles, which were computed from the FFQ (food frequency questionnaire)-derived dietary data. The pooled DII reflects the pro-inflammatory or anti-inflammatory potential of a person’s daily diet. In this study, DII was firstly analyzed as a continuous variable, and then, all participants were categorized into quantiles according to DII (Q1: DII <0.23, Q2: 0.23 ≤ DII < 1.76, Q3: 1.76 ≤ DII < 2.95, Q4: DII ≥2.95). The Healthy Eating Index (HEI) was also calculated in our study, which is another widely used dietary measurement proposed by the United States Department of Agriculture to evaluate dietary quality by comparing the intake of 13 components of one’s daily diet with the Dietary Guidelines for Americans (20). The HEI ranges from 0 to 100; the higher the HEI score, the better the quality of the diet.

### Definition of hypertension

Experienced examiners in the NHANES group recorded the blood pressure of the participants according to the guideline of the American Heart Association (21). The detailed method of the blood pressure measurement can also be found at the NHANES website (http://www.cdc.gov/nchs/nhanes.htm, accessed on 11 November 2022). We obtained the average blood pressure of three consecutive measurements in a calm state. Hypertension was defined as follows: 1) average systolic blood pressure (SBP) ≥140 mmHg, 2) average diastolic blood pressure (DBP) ≥90 mmHg, 3) self-reported hypertension, 4) individuals with prescribed antihypertensive medications. The criteria of 140/90 mmHg refers to the guideline of the International Society of Hypertension (22).

### Covariates

We acquired demographic information such as age, sex, race, and education level from the demographic questionnaire. Ethnic information was presented in alphabetical order. We chose the current ethnic classification method based on the previously published literature. Others include Asian Americans, Native Americans, Latinos, etc. Participants provided the information of smoking status, alcohol consumption, and disease history in the health questionnaire. Blood samples were collected and used to examine the blood biochemical indexes after at least 8 h of an overnight fast. We calculated the eGFR based on the Chronic Kidney Disease Epidemiology Collaboration creatinine equation. For the men: eGFR = (140-age) × body weight (kg) × 1.23/creatinine (μmol/L). For the women: eGFR = (140-age) × body weight (kg) × 1.03/creatinine (μmol/L).

### Statistical analysis

All of the analyses followed the NHANES analytic and reporting guidance. Stratified multistage probability sampling was adopted in the NHANES to reduce the bias caused by post-stratification, nonresponse, and oversampling. According to the primary sampling unit, a specific sampling weight was assigned to each participant to ultimately produce representative estimates nationwide. All analyses produced according to the finally 20-year survey weight in statistical analysis in the present study. Continuous variables were presented as the weighted mean [95% confidence interval (CI)], and categorical variables were represented as proportions (95% CI). Baseline characteristics were compared using the adjusted Wald test for continuous variables and Rao–Scott χ^2^ test for categorical variables. DII components were also compared using the adjusted Wald test. The correlation between DII and HEI was investigated by Spearman method. The weighted multivariable logistic regression analysis was adopted to assess the association of DII with hypertension. Subgroup analyses, stratified by age, sex, body mass index (BMI), and race, were conducted to evaluate the heterogeneity in this study. According to the guideline of the WHO, normal weight was defined as participants with BMI <25 kg/m^2^, overweight was defined as 25 ≤ BMI < 30 kg/m^2^, and obesity was defined as BMI ≥30 kg/m^2^. We carried out LASSO regression based on “*galmet*” package in R. The nomogram model was established based on the “*replot*” package. The discriminatory power of the nomogram model in identifying hypertension risk was evaluated with the receiver operating characteristic (ROC) curve. We adopted “multiple imputation” to fill the missing covariates, avoiding the selection bias due to excluding participants with missing data. A *p* value <0.05 was considered significant. All statistical analyses were conducted using R software (version 4.1.6, R Foundation for Statistical Computing, Vienna, Austria).

## Results

### Characteristics of the study population

**Figure 1:**
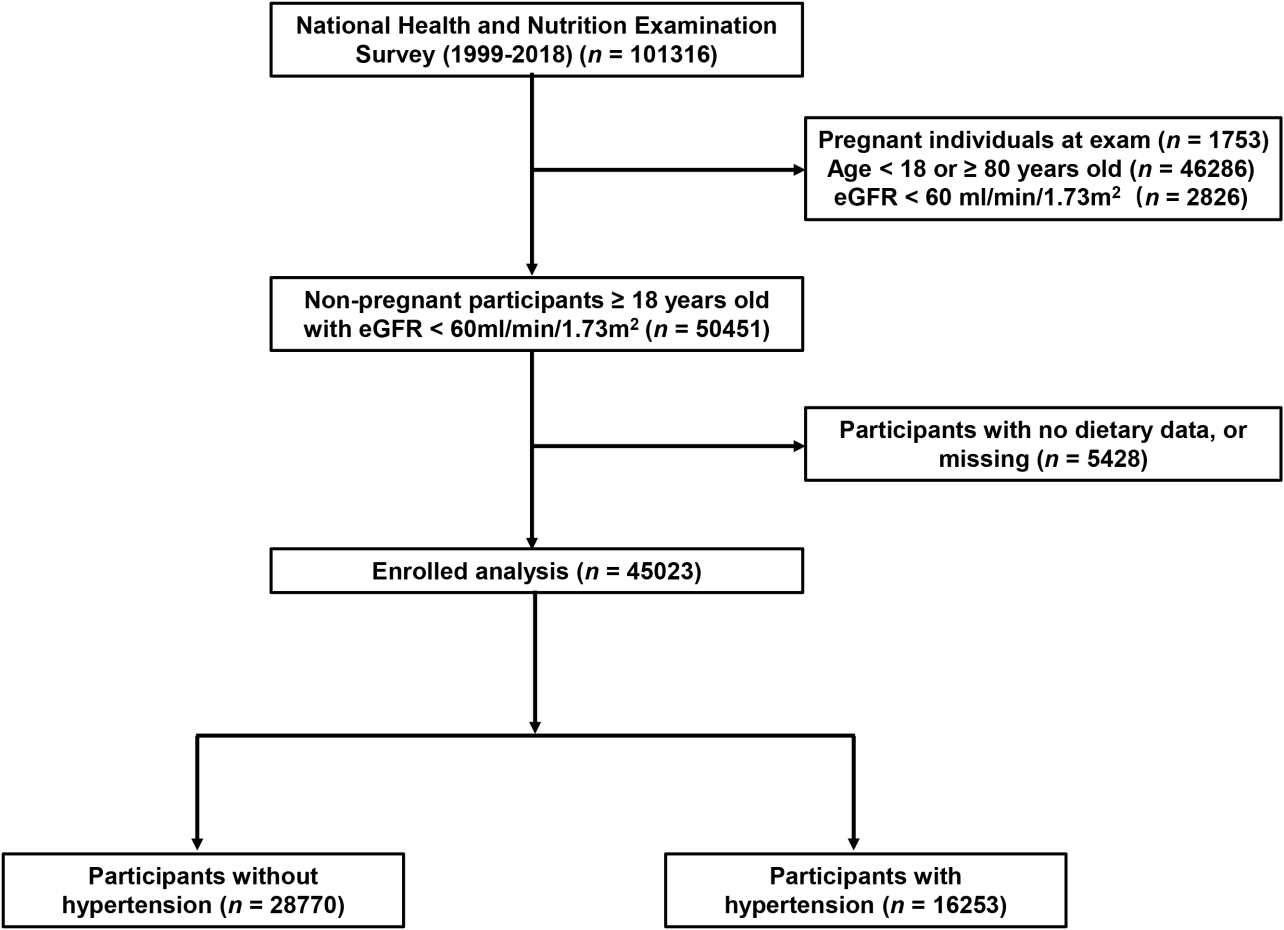
Flowchart of the study population. DII, Dietary Inflammatory Index; eGFR, estimated glomerular filtration rate.

**Figure 2:**
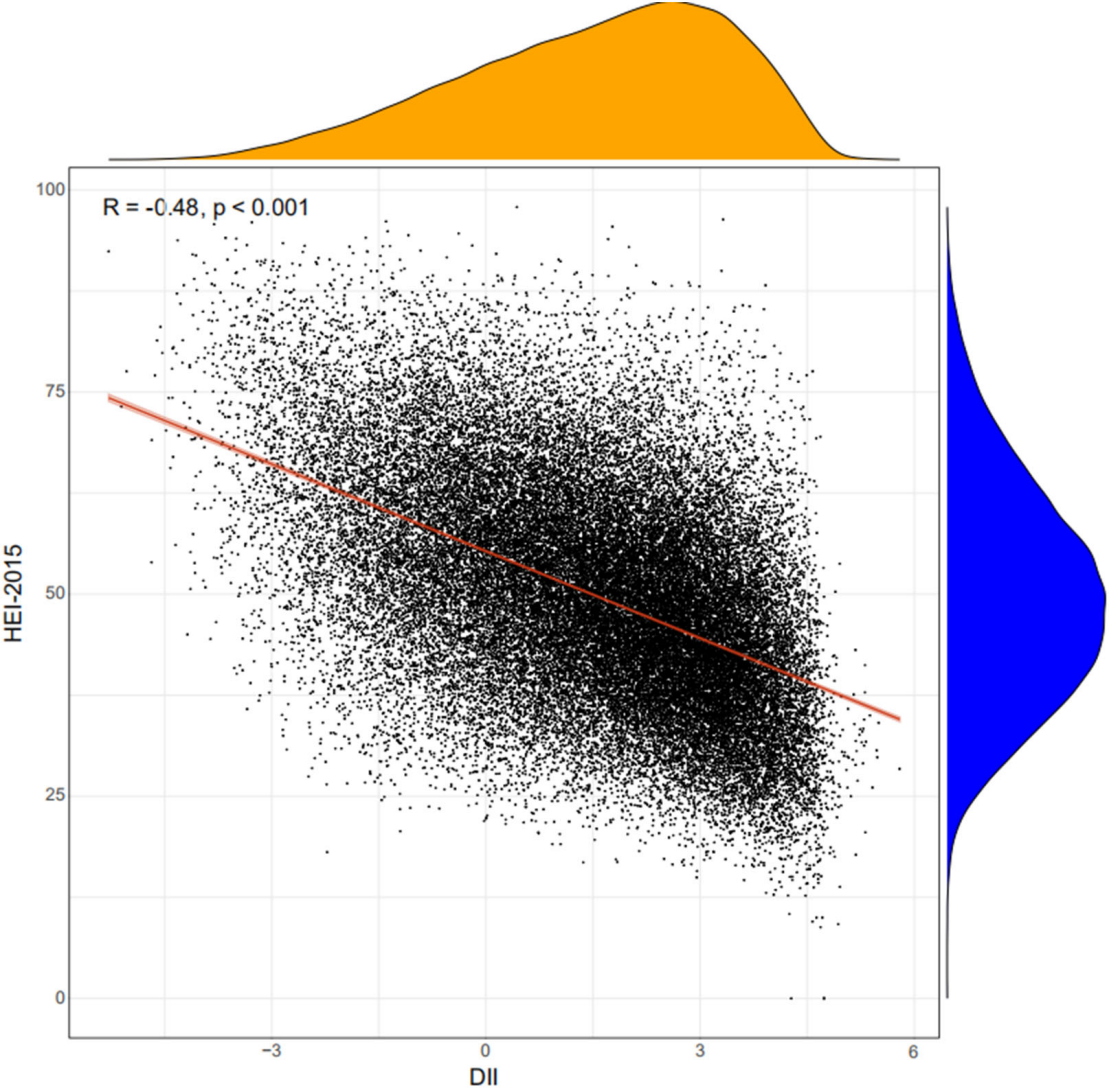
Correlation between DII and HEI. DII, Dietary Inflammatory Index; HEI, Healthy Eating Index.

A total of 45,023 participants from the NHANES (1999–2018) were included in the present study, representing 191 million multiracial adults in the United States, of whom 16,253 (36.1%) had hypertension. Among all participants, 22,516 (49.8%) were men and 18,349 (67.4%) were non-Hispanic white; the mean age of all of the participants was 43.8 years (**Figure 1**). Significant differences were observed in both demographic and clinical characteristics between participants with hypertension and participants without hypertension. Compared with the non-hypertension group, participants in the hypertension group were older (53.2 vs. 39.1 years old, *p* < 0.001), more often men (51.3% vs. 49.0%, *p* < 0.001), with lower education level, and had a higher proportion of smoking and alcohol users. The prevalence rate of diabetes was also higher in participants with hypertension than those without hypertension (21.9% vs. 5.3%, *p* < 0.001). Moreover, the eGFR of the participants with hypertension was significantly lower compared with that of those without hypertension (91.6 vs. 102.2 ml/min/1.73 m^2^, *p* < 0.001). The HEI of the participants with hypertension was lower than that of participants without hypertension (49.6 vs. 50.4, *p* < 0.001). Moreover, we calculated the DII and found that participants in the hypertension group also had a higher mean DII compared with that of participants without hypertension (1.40 vs. 1.32, *p* = 0.002). **Table 1** shows the detailed comparison of baseline characteristics. Similarly, to explore the dietary factors that contributed to the difference of DII between two groups, each component score of DII was presented in **Table 2**. Individuals with hypertension had higher inflammatory scores in protein, dietary fiber, MUFA (monounsaturated fatty acids), PUFA (polyunsatur ated fatty acids), n6 PUFA, cholesterol, vitamin A, vitamin B1, vitamin B2, vitamin B6, vitamin D, vitamin E, folate, niacin, iron, magnesium, zinc, selenium, and alcohol but lower inflammatory scores in energy, carbohydrate, total fatty acid, total saturated fatty acid, n3 polyunsaturated fatty acid, vitamin B12, and caffeine. Baseline characteristics and DII components grouped for all participants grouped by sex were shown in **Table S1** and **Table S2**. The distribution of HEI and DII among the 45,023 participants and the correlation between them were shown in **Figure 2**. We found that HEI had a significant negative correlation with DII (R = -0.48, *p* < 0.001).

**Table 1:** Baseline characteristics of participants enrolled in the sensitivity analysis.

Variables	Overall (*n* = 22,145)	Non-hypertension (*n* = 18,261)	Hypertension (*n* = 3,884)	*p* value
**Age, years**	37.5 (37.2, 37.8)	36.2 (35.8, 36.5)	44.3 (43.8, 44.9)	<0.001***
**Sex, male, *n* (%)**	58.0 (56.1, 60.0)	56.9 (56.1, 57.6)	64.0 (62.0, 66.1)	<0.001***
**Race, *n* (%)**				<0.001***
**Mexican American**	12.64 (11.25, 14.03)	13.13 (11.63, 14.62)	10.21 (8.63, 11.79)	
**Non-Hispanic White**	58.75 (55.13, 62.37)	58.89 (56.65, 61.13)	58.05 (54.71, 61.39)	
**Non-Hispanic Black**	12.98 (11.86, 14.10)	12.15 (10.96, 13.34)	17.13 (15.18, 19.08)	
**Other Hispanic**	7.53 (6.39, 8.66)	7.61 (6.48, 8.74)	7.10 (5.80, 8.41)	
**Other**	8.10 (7.39, 8.82)	8.22 (7.47, 8.97)	7.51 (6.40, 8.61)	
**Smoking, *n* (%)**	25.69 (24.41, 26.97)	24.79 (23.66, 25.91)	30.23 (28.10, 32.36)	<0.001***
**Alcohol users, *n* (%)**	78.26 (75.60, 80.92)	78.45 (77.21, 79.70)	77.28 (75.30, 79.26)	0.21
**Education level, *n* (%)**				<0.001***
**Below high school**	5.74 (5.23, 6.25)	5.55 (5.03, 6.08)	6.68 (5.77, 7.59)	
**High school**	38.67 (36.81, 40.53)	38.06 (36.61, 39.52)	41.72 (39.60, 43.83)	
**Above high school**	55.59 (53.30, 57.88)	56.38 (54.74, 58.03)	51.60 (49.36, 53.84)	
**SBP, mmHg**	118.12 (117.79, 118.45)	114.37 (114.11, 114.63)	137.01 (136.20, 137.82)	<0.001***
**DBP, mmHg**	70.89 (70.58, 71.21)	68.97 (68.68, 69.26)	80.59 (79.94, 81.23)	<0.001***
**Diabetes, *n* (%)**	3.28 (2.96, 3.60)	2.41 (2.13, 2.69)	7.66 (6.54, 8.78)	<0.001***
**FBG, mmol/L**	5.55 (5.53, 5.57)	5.48 (5.46, 5.50)	5.89 (5.81, 5.98)	<0.001***
**HbA1c, %**	5.36 (5.35, 5.38)	5.32 (5.31, 5.33)	5.56 (5.52, 5.61)	<0.001***
**eGFR, ml/min/1.73 m^2^**	104.05 (103.60, 104.50)	105.03 (104.54, 105.52)	99.11 (98.41, 99.81)	0.04*
**TG, mmol/L**	2.08 (2.04, 2.11)	1.99 (1.95, 2.04)	2.49 (2.39, 2.59)	<0.001***
**TC, mmol/L**	5.00 (4.98, 5.02)	4.93 (4.90, 4.95)	5.36 (5.31, 5.42)	<0.001***
**LDL-C, mmol/L**	2.72 (2.70, 2.74)	2.68 (2.66, 2.70)	2.93 (2.88, 2.99)	<0.001***
**HDL-C, mmol/L**	1.34 (1.33, 1.35)	1.35 (1.34, 1.36)	1.31 (1.30, 1.33)	<0.001***
**RBC, ×10^9^/L**	4.83 (4.81, 4.84)	4.81 (4.80, 4.82)	4.90 (4.88, 4.92)	<0.001***
**WBC, ×10^9^/L**	7.19 (7.15, 7.24)	7.15 (7.10, 7.19)	7.43 (7.33, 7.53)	<0.001***
**NE, ×10^9^/L**	4.23 (4.19, 4.26)	4.19 (4.16, 4.23)	4.40 (4.32, 4.47)	<0.001***
**Monocyte, ×10^9^/L**	0.55 (0.55, 0.56)	0.55 (0.55, 0.55)	0.57 (0.56, 0.58)	<0.001***
**LY, ×10^9^/L**	2.17 (2.16, 2.19)	2.16 (2.15, 2.18)	2.20 (2.17, 2.23)	0.03*
**PLT, ×10^6^/L**	255.39 (253.89, 256.88)	254.63 (253.10, 256.17)	259.17 (256.23, 262.10)	0.003**
**Hemoglobin, g/L**	14.58 (14.53, 14.62)	14.53 (14.49, 14.58)	14.79 (14.72, 14.86)	<0.001***
**HEI-2015**	48.99 (48.59, 49.40)	49.07 (48.65, 49.50)	48.60 (47.97, 49.22)	0.14

Continuous variables are presented as the mean and 95% confidence interval, categorical variables are presented as the proportion and 95% confidence interval.

SBP, systolic blood pressure; DBP, diastolic blood pressure; FBG, fasting blood glucose; HbA1c, glycated hemoglobin; eGFR, estimated glomerular filtration rate; BMI, body mass index; WC, waist circumference; TG, triglyceride; TC, total cholesterol; LDL-C, low-density lipoprotein cholesterol; HDL-C, high-density lipoprotein cholesterol; RBC, red blood cell; WBC, white blood cell; NE, neutrophil; LY, lymphocyte; PLT, platelet.

Ethnic information is presented in alphabetical order.

*** p < 0.001, ** p < 0.01, * p < 0.05.

**Table 2:** Comparison of each Component of DII Scores Between Individuals with Hypertension and Individuals without Hypertension.

Variables	Overall(n = 45023)	Non-Hypertension(n = 28770)	Hypertension(n = 16253)	*P* value
**DII**	1.35 (1.30, 1.39)	1.32 (1.27, 1.37)	1.40 (1.35, 1.45)	0.002**
**Energy**	0.00 (0.00, 0.01)	0.01 (0.01, 0.01)	-0.01 (-0.01, 0.00)	<0.001***
**Protein**	0.00 (0.00, 0.00)	0.00 (0.00, 0.00)	0.00 (0.00, 0.00)	<0.001***
**Carbohydrate**	-0.02 (-0.02, -0.01)	-0.01 (-0.01, -0.01)	-0.02 (-0.03, -0.02)	<0.001***
**Dietary fiber**	0.19 (0.18, 0.20)	0.19 (0.18, 0.20)	0.20 (0.19, 0.22)	0.02*
**Total fatty acid**	0.04 (0.03, 0.04)	0.04 (0.04, 0.05)	0.03 (0.02, 0.03)	<0.001***
**Total saturated fatty acid**	-0.06 (-0.06, -0.05)	-0.05 (-0.05, -0.04)	-0.07 (-0.07, -0.06)	<0.001***
**MUFA**	0.00 (0.00, 0.00)	0.00 (0.00, 0.00)	0.00 (0.00, 0.00)	<0.001***
**PUFA**	-0.07 (-0.07, -0.06)	-0.07 (-0.08, -0.07)	-0.06 (-0.06, -0.05)	<0.001***
**n3 Polyunsaturated fatty acid**	0.27 (0.27, 0.27)	0.27 (0.27, 0.27)	0.26 (0.26, 0.27)	0.03*
**n6 Polyunsaturated fatty acid**	-0.06 (-0.06, -0.06)	-0.06 (-0.06, -0.06)	-0.06 (-0.06, -0.06)	<0.001***
**Cholesterol**	-0.02 (-0.02, -0.02)	-0.02 (-0.02, -0.02)	-0.02 (-0.02, -0.02)	0.08
**Vitamin A**	0.19 (0.18, 0.19)	0.19 (0.18, 0.19)	0.19 (0.19, 0.20)	0.01*
**Vitamin B1**	0.01 (0.01, 0.01)	0.01 (0.01, 0.01)	0.02 (0.01, 0.02)	<0.001***
**Vitamin B2**	-0.01 (-0.01, -0.01)	-0.01 (-0.02, -0.01)	-0.01 (-0.01, -0.01)	<0.001***
**Vitamin B6**	-0.09 (-0.09, -0.08)	-0.09 (-0.10, -0.09)	-0.07 (-0.08, -0.07)	<0.001***
**Vitamin B12**	-0.02 (-0.02, -0.02)	-0.02 (-0.02, -0.01)	-0.02 (-0.02, -0.02)	<0.001***
**Vitamin C**	0.19 (0.19, 0.20)	0.19 (0.18, 0.20)	0.20 (0.19, 0.20)	0.1
**Vitamin D**	0.21 (0.20, 0.21)	0.20 (0.19, 0.21)	0.22 (0.21, 0.23)	0.002**
**Vitamin E**	0.07 (0.06, 0.08)	0.07 (0.06, 0.07)	0.07 (0.06, 0.08)	0.19
**Folate**	0.10 (0.10, 0.10)	0.10 (0.10, 0.10)	0.11 (0.11, 0.11)	<0.001***
**β-Carotene**	0.35 (0.34, 0.36)	0.35 (0.34, 0.36)	0.35 (0.34, 0.36)	0.57
**Niacin**	0.02 (0.02, 0.02)	0.02 (0.01, 0.02)	0.03 (0.03, 0.03)	<0.001***
**Iron**	0.00 (0.00, 0.00)	0.00 (0.00, 0.00)	0.00 (0.00, 0.00)	<0.001***
**Magnesium**	0.04 (0.04, 0.05)	0.04 (0.03, 0.04)	0.06 (0.05, 0.06)	<0.001***
**Zinc**	-0.03 (-0.03, -0.02)	-0.03 (-0.04, -0.03)	-0.02 (-0.02, -0.01)	<0.001***
**Selenium**	-0.10 (-0.10, -0.10)	-0.10 (-0.10, -0.10)	-0.10 (-0.10, -0.09)	<0.001***
**Caffeine**	0.08 (0.08, 0.08)	0.08 (0.08, 0.08)	0.08 (0.08, 0.08)	0.003**
**Alcohol**	-0.06 (-0.06, -0.06)	-0.06 (-0.06, -0.06)	-0.06 (-0.06, -0.06)	<0.001***

Data are presented as the mean and 95% confidence interval. DII, dietary inflammatory index; MUFA, monounsaturated fatty acids; PUFA, polyunsaturated fatty acids. *** P value<0.001, ** P value<0.01, * P value<0.05.

### Association of DII with the prevalence of hypertension

**Figure 3:**
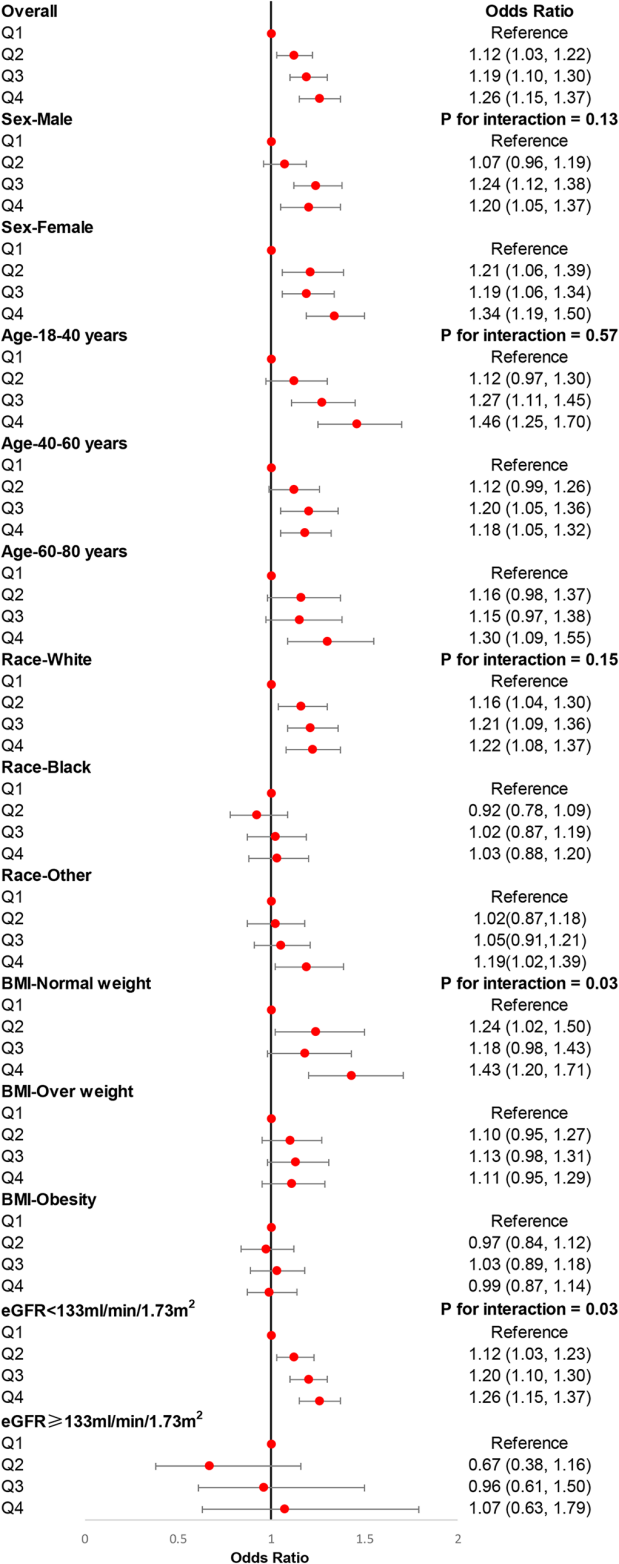
Subgroup analyses for the association of DII with the risk of hypertension. Multivariable weight logistic analyses were conducted among different populations after adjusting for age, sex, race, education level, smoking, alcohol users, diabetes, and eGFR. eGFR, estimated glomerular filtration rate; BMI, body mass index.

Logistic regression was adopted to investigate the association between DII and hypertension in the present study. Our results demonstrated that DII, as a continuous variable, associated with the incidence of hypertension before (OR (odds ratio): 1.02, 95% CI: 1.01–1.04, *p* = 0.002) and after (OR: 1.05, 95% CI: 1.03–1.07, *p* < 0.001) adjusting for age, sex, race, education level, smoking, alcohol users, diabetes, eGFR, and BMI. Then, all participants were divided into quantiles according to the levels of DII, and we found that individuals with higher DII were associated with a higher risk of hypertension compared with individuals with lower DII (**Table 3**). We also carried out subgroup analyses stratified by sex, age (18–40, 40–60, 60–80 years old), race, BMI (normal weight, overweight, obesity), and eGFR (<133 ml/min/1.73 m^2^, ≥133 ml/min/1.73 m^2^) to further explore the association between DII and hypertension among different populations. There was no difference in the association between DII and hypertension in male participants and female participants, either in the age strata, which demonstrated that the conclusion of the present study was stable. However, White participants were more sensitive to DII than other races. Moreover, we did not observe an association between DII and hypertension among overweight participants, participants with obesity, and participants with eGFRs ≥133 ml/min/1.73 m^2^ (**Figure 3**).

**Table 3:** Logistic regression analysis on the association between DII and hypertension.

	Non-adjusted model		Model I		Model II	
	OR [95% CI]	*p* value	OR [95% CI]	*p* value	OR [95% CI]	*p* value
**DII**	1.02 [1.01, 1.04]	0.002**	1.07 [1.05, 1.09]	<0.001***	1.05 [1.03, 1.07]	<0.001***
**Q1**	Reference	–	Reference	–	Reference	–
**Q2**	1.07 [1.00, 1.14]	0.06	1.15 [1.05, 1.25]	0.002**	1.12 [1.03, 1.22]	<0.001***
**Q3**	1.09 [1.02, 1.17]	0.02*	1.25 [1.15, 1.35]	<0.001***	1.19 [1.10, 1.30]	<0.001***
**Q4**	1.10 [1.02, 1.19]	0.01*	1.36 [1.24, 1.48]	<0.001***	1.26 [1.15, 1.37]	<0.001***

Data are presented as OR [95% CI]. Model I adjusted for age, sex, and race/ethnicity. Model II adjusted for age, sex, race/ethnicity, education levels, smoking, alcohol users, diabetes, eGFR, and BMI.

OR, odds ratio; CI, confidence interval; DII, Dietary Inflammatory Index; Q1, first quartile; Q2, second quartile; Q3, third quartile; Q4, fourth quartile. –, not applicable.

*** p < 0.001, ** p < 0.01, * p < 0.05.

### LASSO (least absolute shrinkage and selection operator) regression and the nomogram model

**Figure 4:**
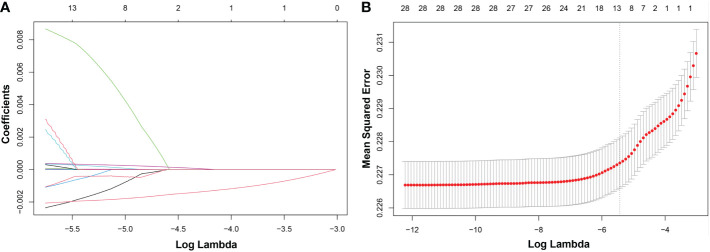
LASSO regression analysis to screen key dietary factors most related to hypertension. **(A)** Plot for LASSO regression coefficients. **(B)** Cross-validation plot. LASSO, least absolute shrinkage and selection operator.

**Figure 5:**
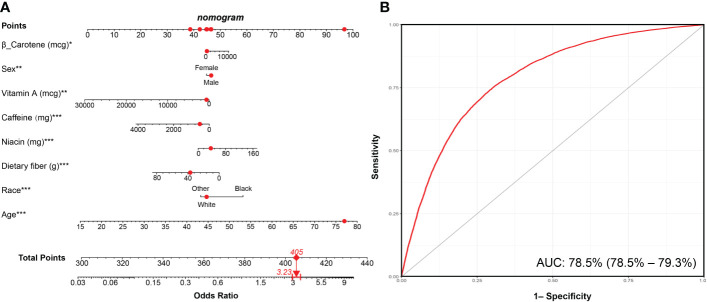
Nomogram model established for predicting the risk of hypertension and its ROC curve. **(A)** Nomogram model based on the key dietary factors screened by LASSO regression, and the red points show an example. For 77-year-old male participants with hypertension, the probability of hypertension increased by 3.23-fold. **(B)** ROC curve for evaluating the diagnostic power of the nomogram model in this study. *** p < 0.001, ** p < 0.01, * p < 0.05.

LASSO regression was adopted to screen the key dietary factors mostly related to hypertension. All of the 28 dietary components that contributed to DII were included in the original LASSO regression model; in addition, three important demographic variables (age, sex, and race) were also included. We selected the penalty parameter lambda (λ) based on 10-fold cross-validation (**Figure 4A**). In this study, 1,000 iterations of the algorithm were performed to ensure accuracy. The same as previous published studies, the largest λ within one standard error range of the minimal binomial deviation (λ = 0.005258) was chosen as the best model (**Figure 4B**). A total of 13 variables (age, sex, race, protein, carbohydrate, dietary fiber, total saturated fat, cholesterol, vitamin A, β-carotene, niacin, vitamin C, caffeine) was used to establish the crude nomogram model, and eight of them (age, sex, race, dietary fiber, vitamin A, β-carotene, niacin, caffeine) that contributed to the model with statistical significance were finally included (**Figure 5A**). Results of the ROC curve showed that the nomogram model established in this study had a favorable discriminatory power, with an area under the curve (AUC) of 78.5% (95% CI: 78.5%–79.3%) (**Figure 5B**).

### Sensitivity analysis

We also carried out a sensitivity analysis in which participants who received any drug treatment were excluded because the application of medication can influence blood pressure. A total of 22,145 participants were included in the sensitivity analysis. **Table S3** shows the detailed demographic and clinical characteristics of these participants. Results of the sensitivity analysis were consistent with those in the main analysis. DII, whether as a continuous variable or a categorical variable, was positively associated with hypertension in the sensitivity analysis. In the fully adjusted logistic regression model, DII was positively associated with hypertension as a continuous variable (OR: 1.05, 95% CI: 1.03–1.08), participants in higher quartiles determined by DII were more likely to have hypertension (Q2: OR: 1.05, 95% CI: 1.03–1.08; Q3: OR: 1.25, 95% CI: 1.09–1.44; Q4: OR: 1.26, 95% CI: 1.10–1.45) (**Table 4**). Therefore, results of the sensitivity analysis demonstrated that the conclusions drawn in the present study are stable and reliable.

**Table 4:** Logistic regression analysis on the association between DII and hypertension in the sensitivity analysis.

	Non-adjusted model		Model I		Model II	
	OR [95% CI]	*p* value	OR [95% CI]	*p* value	OR [95% CI]	*p* value
**DII**	1.03 [1.00, 1.05]	0.05*	1.07 [1.05, 1.10]	<0.001***	1.05 [1.03, 1.08]	<0.001***
**Q1**	Reference	–	Reference	–	Reference	–
**Q2**	1.03 [0.90, 1.18]	0.62	1.10 [0.95, 1.27]	0.21	1.07 [0.92, 1.23]	<0.39
**Q3**	1.14 [1.00, 1.29]	0.05*	1.33 [1.16, 1.52]	<0.001***	1.25 [1.09, 1.44]	0.002**
**Q4**	1.10 [0.97, 1.26]	0.15	1.37 [1.19, 1.58]	<0.001***	1.26 [1.10, 1.45]	0.001**

Data are presented as OR [95% CI]. Model I adjusted for age, sex, and race/ethnicity. Model II adjusted for age, sex, race/ethnicity, education levels, smoking, alcohol users, diabetes, eGFR, and BMI.

OR, odds ratio; CI, confidence interval; DII, Dietary Inflammatory Index; Q1, first quartile; Q2, second quartile; Q3, third quartile; Q4, fourth quartile. –, not applicable.

*** p < 0.001, ** p < 0.01, * p < 0.05.

## Discussion

A large number of studies have already demonstrated that dietary inflammation potential associated with hypertension; however, the association of DII with hypertension still needs further investigation. In this weighted cross-sectional study based on the NHANES, 45,023 residents in the United States were included to study the association of DII with hypertension, representing 191 million adults in the United States. The main and most important findings are as follows: 1) participants with hypertension had a higher mean DII than those without hypertension; 2) DII negatively correlated with HEI in this large population; 3) an increment of DII strongly associated with the risk of hypertension after adjusting for confounding factors; 4) dietary fiber, vitamin A, β-carotene, niacin, and caffeine are the key dietary factors mostly associated with hypertension; 5) a nomogram model established in this study based on key dietary factors showed a favorable diagnostic power in identifying hypertension risk.

The role of dietary habits in systemic inflammation has been extensively explored; evidence showed that Western dietary patterns, characterized by high fat and high calorie content, can lead to increased systemic inflammation (23). Adherence to Western dietary patterns can lead to hyperlipidemia and hyperglycemia, and it can also activate nonenzymatic glycosylation and glucose-induced NADH (Nicotinamide adenine dinucleotide), generating excessive reactive oxygen species (ROS), eventually causing oxidative stress and increased systemic inflammation levels (24). Moreover, a recent animal study reported that Western dietary patterns disrupt the gut microbial ecosystem and promote chronic intestinal inflammation (25). Results of a recent meta-analysis that pooled 22 high-quality randomized clinical trials showed that dietary patterns had a great impact on blood inflammatory markers, with the Western diet significantly increasing the levels of IL-1β, IL-6, and CRP, whereas the Mediterranean diet had anti-inflammation effects (26). DII is a novel measurement designed to evaluate dietary inflammation potential as previously described. In this study, we compared the DII and its 28 components between participants with and without hypertension. We found that participants with hypertension had an elevated mean DII compared with that of participants without hypertension. As DII is a novel measurement, we also calculated HEI for all participants and explored the correlation between DII and HEI in a large population. HEI is a widely used index to evaluate dietary quality, and we found that DII had a significant negative association with HEI. Dietary inflammation potential has a close association with various cardiovascular diseases. It has been revealed that unhealthy eating habits can activate the inflammasome complex, which plays an important role in the pathology of CHD, arterial stiffness, hypertension, etc. (27). Therefore, improving dietary quality is helpful for the prevention of not only hypertension but also other cardiovascular diseases.

Hypertension is a very common clinical syndrome characterized by elevated SBP/DBP (28). The molecular mechanisms of hypertension still remain unclear; inflammation plays a crucial role in the occurrence and development of hypertension (29). Jayedi et al. (30) assessed the relationship between inflammatory markers and hypertension risk in a recent study, and they found that CRP and IL-6, but not IL-1β, had a favorable predictive ability in hypertension risk. Moreover, researchers also found that dietary approaches to stop hypertension (DASH) diet is associated with not only improved blood pressure control but also decreased systemic inflammatory markers in adults compared with usual diets (31, 32). It is well-known that hypertension closely associates with arterial stiffness in the elderly, with arterial stiffness being the main reason for isolated systolic hypertension (33, 34). Arterial stiffness is mostly due to vascular remodeling, and the main manifestations of vascular remodeling were vascular wall thickening, increased wall/cavity ratio, reduced number of microarteries, and the consequent abnormal vascular function (22, 35, 36). Inflammation is involved in the process of arterial stiffness, and inhibition of inflammation can attenuate vascular remodeling, eventually reducing hypertension risk (37–39). Therefore, it is necessary to establish a measurement to evaluate the dietary inflammation potential. In this study, we assessed the association of DII with the risk of hypertension. Our results showed that an increment of DII closely associated with the prevalence of hypertension. In a previous study focused on the association of DII with hypertension, investigators included 47,548 female participants from a French cohort (E3N cohort) and found that there was a weak association between DII and the risk of hypertension. The conclusion drawn in this study may be due to the study including only female patients, but the incidence rate of hypertension is higher in male individuals. Moreover, this study did not conduct a subgroup analysis to explore the relationship between DII and hypertension in different populations. Compared with the study based on the E3N cohort, we included a similar number of participants (45,023 vs. 47,548), of whom 22,516 (49.8%) were male participants and 22,507 (50.2%) were female individuals. We found a significant association of DII with hypertension in the patients from the NHANES (40). Shoaei et al. (41) recently carried out a case-control study on 945 middle-aged participants (only 40–60-year-old participants), and the investigators found that DII had a close association with the occurrence of hypertension in the middle-aged population. The conclusions drawn in this study were similar to those of our subgroup analysis. However, due to the small sample size and the inclusion of only middle-aged participants, it is necessary to explore the relationship between DII and hypertension in a larger and more general population (41). In the sensitivity analysis of our study, participants with any medication treatment were excluded to investigate the association of DII with hypertension in a more general population, and the results were consistent with the main analysis. Considering that the application of DII in the hypertension field is still limited at present, investigators should pay more attention to DII for preventing hypertension and predicting the prognosis of patients with hypertension.

Although DII has been validated that it can reflect the dietary inflammation potential and systemic inflammation level, it is not designed for predicting hypertension (42, 43). We aimed at screening dietary factors most related to the risk of hypertension. Therefore, LASSO regression analysis was adopted, and we found that protein, carbohydrate, dietary fiber, total saturated fat, cholesterol, vitamin A, β-carotene, niacin, vitamin C, and caffeine were key dietary components closely associated with hypertension. Dietary fiber, vitamin A, β-carotene, niacin, and caffeine were the final dietary components included in the LASSO-logistic regression model due to their statistically significant contribution to the predicting model. It is of interest to note that the relationship between the key dietary factors screened in this study and hypertension has been investigated in both clinical and animal studies. Results of a recent cross-sectional study also based on the NHANES showed that dietary fiber intake negatively correlated with the prevalence of hypertension; moreover, uncontrolled hypertension associated with poorer cognitive function in elderly participants with low, but not high, dietary fiber intake (44). Another prospective study enrolled 12,245 participants who were free of hypertension at baseline from the China Health and Nutrition Survey (CHNS) and showed an L-shaped relation of dietary vitamin A intake with the incidence of new-onset hypertension in general Chinese adults (45). Yanagisawa et al. (46) found that β-carotene had an impact on IL-1β C-31T polymorphism; moreover, IL-1β C-31T polymorphism positively correlated with the incidence of hypertension, and this relationship was modulated by serum β-carotene levels (46). Vitamin C can inhibit oxidative stress and attenuate vascular remodeling, and it has been reported that it prevents hypertension in an animal model induced by heat exposure. A prospective study that included 1,204 participants showed that coffee consumption increased the risk of hypertension after a median follow-up of 12.6 years (47). In the present study, we established a nomogram model based on these key dietary factors and with a favorable diagnostic power in ROC curve analysis, which can be helpful for the prevention and treatment of hypertension.

There are several advantages and limitations of this study. First, a reliable conclusion can be drawn due to the large population included in this study. All of the statistics followed the guidelines of the NHANES; therefore, the conclusion in this study can be applied to the 191 million residents in the United States. Second, we carried out LASSO regression analysis to screen key dietary factors mostly related to hypertension and established a nomogram model with a favorable discriminative power. Some limitations of this present study also have to be pointed out. First, we can only draw correlation conclusions because this study is a cross-sectional study. Because the application of DII in the hypertension field is limited, more prospective studies are needed. Second, there may be a subjective bias due to the self-reported dietary information and covariates acquired from the NHANES questionnaires. Moreover, as we all know, one’s daily diet changes a lot, and a 24-h recall of dietary information may be insufficient. Third, there are large ethnic differences in diet, physical activity, genetic variants, lipid metabolism, and susceptibility to cardiovascular disease. Therefore, whether the conclusion in the present study based on US participants is applicable to other populations needs to be further explored in future works.

## Conclusions

We retrospectively analyzed 45,023 participants from the NHANES and found that an increment of DII closely associated with the risk of hypertension. The nomogram model established in this study based on key dietary factors screened by LASSO regression showed a favorable diagnostic power in predicting hypertension risk. More attention should be paid to controlling dietary inflammation to prevent and treat hypertension, and more prospective studies are still needed.

## Data availability statement

The datasets presented in this study can be found in online repositories. The names of the repository/repositories and accession number(s) can be found in the article/**Supplementary Material**.

## Ethics statement

This study was approved by National Center for Health Statistics Research Ethics Review Board. The participants provided informed consent to participate in the NHANES. The patients/participants provided their written informed consent to participate in this study.

## Author contributions

L-DW and JL were involved in the experiment design. NZ and L-DW performed the data analysis. L-DW, NZ, and Z-PX wrote the manuscript. QL, YX, J-YW, and S-CD reviewed the manuscript and provided critical suggestions. J-YW received National Natural Science Foundation to help disseminate the findings of this study. All authors contributed to the article and approved the submitted version.
