# Supplementary material for: The dietary inflammatory index and its association with the prevalence of hypertension: A cross-sectional study
Source: Front Immunol. 2023 Jan 18;13:1097228. doi: 10.3389/fimmu.2022.1097228 (PMC9893776; doi:10.3389/fimmu.2022.1097228)
Supplement: Supplementary file 1 [file Table_1.docx]

**Table S1.** **Baseline Characteristics Grouped by Sex.**

| Variables | Male | | | | Female | | | | *P* value between male and female |
| --- | --- | --- | --- | --- | --- | --- | --- | --- | --- |
|  | **Overall**  **(*n* = 22516)** | **Non-hypertension**  **(*n* = 14285)** | **Hypertension**  **(*n* = 8231)** | ***P* value** | **Overall**  **(*n* = 22507)** | **Non-hypertension**  **(*n* = 14485)** | **Hypertension**  **(n = 8022)** | ***P* value** |  |
| Age, years | 43.2 (42.8, 43.6) | 38.8 (38.4, 39.2) | 51.7 (51.2, 52.1) | <0.001*** | 44.4 (44.1, 44.8) | 39.5 (39.1, 39.81) | 54.9 (54.5, 55.3) | <0.001*** | <0.001*** |
| Race, *n* (%) |  |  |  | <0.001*** |  |  |  | <0.001*** | <0.001*** |
| Mexican American | 9.34 (8.28, 10.40) | 10.98 (9.71, 12.26) | 6.18 (5.13, 7.23) |  | 7.95 (6.99, 8.90) | 8.91 (7.77, 10.05) | 5.91 (4.94, 6.89) |  |  |
| Non-Hispanic White | 67.83 (63.81, 71.84) | 66.27 (64.20, 68.34) | 70.82 (68.54, 73.10) |  | 67.01 (62.81, 71.22) | 67.25 (65.11, 69.39) | 66.52 (63.96, 69.08) |  |  |
| Non-Hispanic Black | 10.45 (9.58, 11.32) | 9.53 (8.57, 10.48) | 12.24 (10.89, 13.59) |  | 12.11 (11.00, 13.21) | 10.09 (9.04, 11.14) | 16.36 (14.50, 18.22) |  |  |
| Other Hispanic | 5.59 (4.74, 6.43) | 6.15 (5.18, 7.12) | 4.51 (3.70, 5.32) |  | 6.07 (5.18, 6.97) | 6.54 (5.54, 7.55) | 5.09 (4.11, 6.06) |  |  |
| Other | 6.79 (6.17, 7.41) | 7.08 (6.38, 7.77) | 6.25 (5.44, 7.05) |  | 6.86 (6.26, 7.46) | 7.21 (6.49, 7.92) | 6.12 (5.33, 6.91) |  |  |
| Smoking, *n* (%) | 25.64 (24.38, 26.89) | 26.90 (25.69, 28.10) | 23.21 (21.99, 24.44) | <0.001*** | 20.34 (19.18, 21.50) | 20.78 (19.73, 21.83) | 19.43 (18.28, 20.57) | 0.07 | <0.001*** |
| Alcohol users, *n* (%) | 80.15 (77.33, 82.96) | 81.65 (80.52, 82.77) | 77.26 (75.68, 78.85) | <0.001*** | 71.55 (68.60, 74.50) | 74.81 (73.43, 76.19) | 64.68 (62.78, 66.59) | <0.001*** | <0.001*** |
| Education level, *n* (%) |  |  |  | 0.06 |  |  |  | <0.001*** | <0.001*** |
| Below high school | 5.46 (5.01, 5.90) | 5.15 (4.67, 5.63) | 6.05 (5.37, 6.72) |  | 4.87 (4.48, 5.26) | 4.02 (3.63, 4.41) | 6.67 (5.91, 7.43) |  |  |
| High school | 38.05 (35.95, 40.14) | 38.30 (36.71, 39.88) | 37.57 (35.84, 39.30) |  | 34.67 (32.99, 36.35) | 32.29 (30.84, 33.74) | 39.70 (38.29, 41.10) |  |  |
| Above high school | 56.50 (54.12, 58.87) | 56.55 (54.84, 58.27) | 56.38 (54.53, 58.24) |  | 60.46 (57.74, 63.17) | 63.70 (62.12, 65.27) | 53.63 (52.12, 55.15) |  |  |
| SBP, mmHg | 122.63 (122.29, 122.98) | 117.21 (116.94, 117.48) | 133.06 (132.46, 133.66) | <0.001*** | 118.88 (118.51, 119.25) | 111.98 (111.70, 112.26) | 133.43 (132.82, 134.03) | <0.001*** | <0.001*** |
| DBP, mmHg | 72.79 (72.48, 73.09) | 70.31 (70.01, 70.62) | 77.54 (77.07, 78.01) | <0.001*** | 70.20 (69.90, 70.51) | 68.27 (67.96, 68.57) | 74.29 (73.83, 74.75) | <0.001*** | <0.001*** |
| Diabetes, *n* (%) | 11.37 (10.68, 12.06) | 5.84 (5.26, 6.42) | 22.00 (20.77, 23.23) | <0.001*** | 10.30 (9.65, 10.94) | 4.86 (4.43, 5.29) | 21.76 (20.54, 22.97) | <0.001*** | 0.01* |
| FBG, mmol/L | 5.91 (5.88, 5.95) | 5.69 (5.66, 5.72) | 6.34 (6.28, 6.40) | <0.001*** | 5.66 (5.63, 5.68) | 5.44 (5.41, 5.46) | 6.12 (6.07, 6.17) | <0.001*** | <0.001*** |
| HbA1c, % | 5.55 (5.53, 5.57) | 5.41 (5.40, 5.43) | 5.81 (5.78, 5.85) | <0.001*** | 5.50 (5.49, 5.52) | 5.36 (5.34, 5.37) | 5.82 (5.79, 5.85) | <0.001*** | <0.001*** |
| eGFR, ml/min/1.73m^2^ | 97.90 (97.47, 98.33) | 101.04 (100.55, 101.54) | 91.85 (91.30, 92.40) | <0.001*** | 99.46 (98.92, 99.99) | 103.33 (102.71, 103.95) | 91.28 (90.72, 91.85) | <0.001*** | <0.001*** |
| TG, mmol/L | 2.24 (2.20, 2.28) | 2.16 (2.11, 2.21) | 2.39 (2.32, 2.46) | <0.001*** | 1.99 (1.95, 2.03) | 1.87 (1.83, 1.91) | 2.24 (2.17, 2.30) | <0.001*** | <0.001*** |
| TC, mmol/L | 5.01 (4.98, 5.03) | 4.96 (4.94, 4.99) | 5.09 (5.06, 5.13) | <0.001*** | 5.10 (5.08, 5.13) | 5.00 (4.98, 5.02) | 5.32 (5.29, 5.36) | <0.001*** | <0.001*** |
| LDL-C, mmol/L | 2.77 (2.75, 2.79) | 2.75 (2.73, 2.78) | 2.80 (2.77, 2.83) | 0.01* | 2.70 (2.68, 2.72) | 2.64 (2.61, 2.66) | 2.83 (2.80, 2.86) | <0.001*** | <0.001*** |
| HDL-C, mmol/L | 1.23 (1.22, 1.24) | 1.24 (1.23, 1.25) | 1.21 (1.20, 1.22) | <0.001*** | 1.50 (1.49, 1.51) | 1.51 (1.50, 1.53) | 1.48 (1.47, 1.49) | <0.001*** | <0.001*** |
| RBC, ×10^9^/L | 5.01 (4.99, 5.02) | 5.02 (5.01, 5.04) | 4.97 (4.95, 4.99) | <0.001*** | 4.49 (4.48, 4.50) | 4.48 (4.46, 4.49) | 4.52 (4.50, 4.53) | <0.001*** | <0.001*** |
| WBC, ×10^9^/L | 7.18 (7.13, 7.23) | 7.07 (7.01, 7.12) | 7.39 (7.31, 7.46) | <0.001*** | 7.31 (7.26, 7.36) | 7.25 (7.20, 7.30) | 7.43 (7.35, 7.51) | <0.001*** | <0.001*** |
| NE, ×10^9^/L | 4.22 (4.19, 4.26) | 4.13 (4.09, 4.17) | 4.41 (4.35, 4.46) | <0.001*** | 4.34 (4.30, 4.38) | 4.31 (4.27, 4.35) | 4.40 (4.34, 4.46) | 0.002** | <0.001*** |
| Monocyte, ×10^9^/L | 0.59 (0.58, 0.59) | 0.58 (0.57, 0.58) | 0.61 (0.60, 0.62) | <0.001*** | 0.53 (0.53, 0.53) | 0.52 (0.52, 0.53) | 0.55 (0.54, 0.55) | <0.001*** | <0.001*** |
| LY, ×10^9^/L | 2.11 (2.09, 2.12) | 2.11 (2.09, 2.13) | 2.10 (2.07, 2.13) | 0.44 | 2.21 (2.19, 2.22) | 2.19 (2.17, 2.21) | 2.24 (2.21, 2.27) | 0.005** | <0.001*** |
| PLT, ×10^6^/L | 240.95 (239.63, 242.27) | 242.20 (240.82, 243.57) | 238.56 (236.41, 240.71) | 0.001** | 270.58 (268.96, 272.21) | 268.97 (267.22, 270.73) | 273.97 (271.62, 276.32) | <0.001*** | <0.001*** |
| Hemoglobin, g/L | 15.29 (15.25, 15.33) | 15.33 (15.29, 15.38) | 15.21 (15.16, 15.26) | <0.001*** | 13.52 (13.48, 13.56) | 13.48 (13.44, 13.52) | 13.60 (13.54, 13.65) | <0.001*** | <0.001*** |

Continuous variables are presented as the mean and 95% confidence interval, category variables are presented as the proportion and 95% confidence interval. SBP, systolic blood pressure; DBP, diastolic blood pressure; FBG, fasting blood glucose; HbA1c, glycated hemoglobin; eGFR, estimated glomerular filtration rate; BMI, body mass index; WC, waist circumference; TG, triglycerides; TC, total cholesterol; LDL-C, low-density lipoprotein cholesterol; HDL-C, high-density lipoprotein cholesterol; RBC, red blood cells; WBC, white blood cells; NE, neutrophils; LY, lymphocytes; PLT, platelets. Ethic information is presented in alphabetical order. *** P value<0.001, ** P value<0.01, * P value<0.05.
