# Supplementary material for: The dietary inflammatory index and its association with the prevalence of hypertension: A cross-sectional study
Source: Front Immunol. 2023 Jan 18;13:1097228. doi: 10.3389/fimmu.2022.1097228 (PMC9893776; doi:10.3389/fimmu.2022.1097228)
Supplement: Supplementary file 2 [file Table_2.docx]

**Table S2.** **Comparison of Each Component of DII Scores of All Participants Grouped by Sex.**

| Variables | Male | | | | Female | | | | *P* value between male and female |
| --- | --- | --- | --- | --- | --- | --- | --- | --- | --- |
|  | **Overall**  **(n = 22516)** | **Non-hypertension**  **(n = 14285)** | **Hypertension**  **(n = 8231)** | ***P* value** | **Overall**  **(n = 22507)** | **Non-hypertension**  **(n = 14485)** | **Hypertension**  **(n = 8022)** | ***P* value** |  |
| DII | 0.97 (0.93, 1.02) | 0.93 (0.88, 0.98) | 1.05 (0.99, 1.11) | 0.001** | 1.72 (1.67, 1.77) | 1.69 (1.63, 1.75) | 1.78 (1.72, 1.84) | 0.01* | <0.001*** |
| Energy | 0.06 (0.06, 0.06) | 0.07 (0.06, 0.07) | 0.05 (0.04, 0.05) | <0.001*** | -0.05 (-0.05, -0.05) | -0.04 (-0.05, -0.04) | -0.06 (-0.07, -0.06) | <0.001*** | <0.001*** |
| Protein | 0.01 (0.01, 0.01) | 0.01 (0.01, 0.01) | 0.00 (0.00, 0.00) | <0.001*** | -0.01 (-0.01, -0.01) | -0.01 (-0.01, -0.01) | -0.01 (-0.01, -0.01) | <0.001*** | <0.001*** |
| Carbohydrate | 0.01 (0.01, 0.01) | 0.01 (0.01, 0.02) | 0.00 (-0.01, 0.00) | <0.001*** | -0.04 (-0.04, -0.04) | -0.04 (-0.04, -0.04) | -0.05 (-0.05, -0.04) | <0.001*** | <0.001*** |
| Dietary fiber | 0.11 (0.10, 0.12) | 0.10 (0.09, 0.12) | 0.13 (0.11, 0.14) | 0.01* | 0.28 (0.26, 0.29) | 0.27 (0.26, 0.29) | 0.28 (0.27, 0.30) | 0.11 | <0.001*** |
| Total fatty acid | 0.10 (0.10, 0.10) | 0.11 (0.10, 0.11) | 0.09 (0.08, 0.10) | 0.001** | -0.03 (-0.03, -0.02) | -0.02 (-0.02, -0.02) | -0.04 (-0.04, -0.03) | <0.001*** | <0.001*** |
| Total saturated fatty acid | 0.02 (0.02, 0.03) | 0.03 (0.02, 0.04) | 0.01 (0.00, 0.01) | <0.001*** | -0.13 (-0.14, -0.13) | -0.13 (-0.13, -0.12) | -0.15 (-0.15, -0.14) | <0.001*** | <0.001*** |
| MUFA | 0.00 (0.00, 0.00) | 0.00 (0.00, 0.00) | 0.00 (0.00, 0.00) | 0.004** | 0.00 (0.00, 0.00) | 0.00 (0.00, 0.00) | 0.00 (0.00, 0.00) | <0.001*** | <0.001*** |
| PUFA | -0.12 (-0.12, -0.12) | -0.12 (-0.13, -0.12) | -0.11 (-0.12, -0.11) | 0.03* | -0.01 (-0.02, -0.01) | -0.02 (-0.03, -0.01) | 0.00 (-0.01, 0.00) | <0.001*** | <0.001*** |
| n3 Polyunsaturated fatty acid | 0.26 (0.26, 0.26) | 0.26 (0.26, 0.26) | 0.26 (0.26, 0.26) | 0.24 | 0.27 (0.27, 0.27) | 0.27 (0.27, 0.27) | 0.27 (0.27, 0.27) | 0.07 | <0.001*** |
| n6 Polyunsaturated fatty acid | -0.08 (-0.08, -0.08) | -0.08 (-0.08, -0.08) | -0.08 (-0.08, -0.07) | 0.02* | -0.04 (-0.04, -0.04) | -0.04 (-0.05, -0.04) | -0.04 (-0.04, -0.04) | <0.001*** | <0.001*** |
| Cholesterol | 0.00 (0.00, 0.00) | 0.00 (0.00, 0.00) | 0.00 (0.00, 0.01) | 0.25 | -0.04 (-0.04, -0.04) | -0.04 (-0.04, -0.04) | -0.04 (-0.04, -0.04) | 0.65 | <0.001*** |
| Vitamin A | 0.17 (0.17, 0.18) | 0.17 (0.16, 0.17) | 0.18 (0.17, 0.18) | 0.002** | 0.20 (0.20, 0.21) | 0.20 (0.20, 0.21) | 0.21 (0.20, 0.21) | 0.27 | <0.001*** |
| Vitamin B1 | -0.01 (-0.01, -0.01) | -0.01 (-0.01, -0.01) | 0.00 (0.00, 0.00) | <0.001*** | 0.03 (0.03, 0.03) | 0.03 (0.03, 0.03) | 0.03 (0.03, 0.04) | 0.001** | <0.001*** |
| Vitamin B2 | -0.03 (-0.03, -0.02) | -0.03 (-0.03, -0.02) | -0.02 (-0.02, -0.02) | <0.001*** | 0.00 (0.00, 0.00) | 0.00 (-0.01, 0.00) | 0.00 (0.00, 0.00) | <0.001*** | <0.001*** |
| Vitamin B6 | -0.15 (-0.16, -0.15) | -0.16 (-0.17, -0.16) | -0.14 (-0.14, -0.13) | <0.001*** | -0.02 (-0.03, -0.01) | -0.03 (-0.03, -0.02) | -0.01 (-0.02, 0.00) | <0.001*** | <0.001*** |
| Vitamin B12 | 0.00 (0.00, 0.00) | 0.00 (0.00, 0.00) | 0.00 (-0.01, 0.00) | <0.001*** | -0.03 (-0.03, -0.03) | -0.03 (-0.03, -0.03) | -0.03 (-0.04, -0.03) | 0.23 | <0.001*** |
| Vitamin C | 0.17 (0.17, 0.18) | 0.17 (0.16, 0.18) | 0.18 (0.17, 0.19) | 0.01* | 0.21 (0.20, 0.22) | 0.21 (0.20, 0.22) | 0.21 (0.20, 0.22) | 0.99 | <0.001*** |
| Vitamin D | 0.17 (0.16, 0.18) | 0.16 (0.15, 0.17) | 0.19 (0.18, 0.20) | <0.001*** | 0.24 (0.23, 0.25) | 0.24 (0.23, 0.25) | 0.25 (0.24, 0.26) | 0.1 | <0.001*** |
| Vitamin E | 0.02 (0.01, 0.03) | 0.02 (0.01, 0.02) | 0.02 (0.01, 0.03) | 0.53 | 0.12 (0.11, 0.13) | 0.12 (0.11, 0.13) | 0.13 (0.12, 0.14) | 0.04* | <0.001*** |
| Folate | 0.08 (0.08, 0.08) | 0.07 (0.07, 0.08) | 0.09 (0.09, 0.10) | <0.001*** | 0.12 (0.12, 0.12) | 0.12 (0.12, 0.12) | 0.12 (0.12, 0.13) | 0.08 | <0.001*** |
| β-Carotene | 0.36 (0.36, 0.37) | 0.37 (0.36, 0.38) | 0.36 (0.35, 0.37) | 0.38 | 0.34 (0.33, 0.35) | 0.34 (0.33, 0.35) | 0.34 (0.33, 0.35) | 0.96 | <0.001*** |
| Niacin | -0.03 (-0.03, -0.03) | -0.04 (-0.04, -0.03) | -0.02 (-0.02, -0.01) | <0.001*** | 0.07 (0.07, 0.08) | 0.07 (0.07, 0.07) | 0.08 (0.08, 0.09) | <0.001*** | <0.001*** |
| Iron | 0.01 (0.01, 0.01) | 0.01 (0.01, 0.01) | 0.01 (0.01, 0.01) | <0.001*** | -0.01 (-0.01, 0.00) | 0.00 (-0.01, 0.00) | -0.01 (-0.01, -0.01) | 0.02* | <0.001*** |
| Magnesium | -0.03 (-0.04, -0.03) | -0.04 (-0.05, -0.03) | -0.02 (-0.03, -0.01) | <0.001*** | 0.12 (0.11, 0.13) | 0.11 (0.11, 0.12) | 0.14 (0.13, 0.14) | <0.001*** | <0.001*** |
| Zinc | -0.10 (-0.11, -0.10) | -0.11 (-0.12, -0.11) | -0.09 (-0.10, -0.08) | <0.001*** | 0.05 (0.04, 0.05) | 0.04 (0.04, 0.05) | 0.06 (0.05, 0.07) | 0.001** | <0.001*** |
| Selenium | -0.13 (-0.13, -0.13) | -0.13 (-0.14, -0.13) | -0.13 (-0.13, -0.12) | <0.001*** | -0.07 (-0.07, -0.07) | -0.07 (-0.07, -0.07) | -0.06 (-0.07, -0.06) | 0.002** | <0.001*** |
| Caffeine | 0.08 (0.08, 0.08) | 0.08 (0.08, 0.08) | 0.08 (0.08, 0.08) | <0.001*** | 0.08 (0.08, 0.08) | 0.08 (0.08, 0.08) | 0.08 (0.08, 0.08) | 0.55 | <0.001*** |
| Alcohol | 0.12 (0.11, 0.13) | 0.12 (0.12, 0.13) | 0.11 (0.11, 0.12) | 0.04* | 0.19 (0.19, 0.20) | 0.19 (0.18, 0.19) | 0.20 (0.19, 0.20) | 0.004** | <0.001*** |

Data are presented the mean and 95% confidence interval. DII, dietary inflammatory index; MUFA, monounsaturated fatty acids; PUFA, polyunsaturated fatty acids. *** *P* value<0.001, ** *P* value<0.01, * *P* value<0.05.
