# Supplementary material for: The dietary inflammatory index and its association with the prevalence of hypertension: A cross-sectional study
Source: Front Immunol. 2023 Jan 18;13:1097228. doi: 10.3389/fimmu.2022.1097228 (PMC9893776; doi:10.3389/fimmu.2022.1097228)
Supplement: Supplementary file 3 [file Table_3.docx]

**Table S3. Baseline Characteristics of Participants Enrolled in the Sensitive Analysis.**

| Variables | Overall  (*n* = 22145) | Non-hypertension  (*n* = 18261) | Hypertension  (*n* = 3884) | *P* value |
| --- | --- | --- | --- | --- |
| Age, years | 37.5 (37.2, 37.8) | 36.2 (35.8, 36.5) | 44.3 (43.8, 44.9) | <0.001*** |
| Sex-male, *n* (%) | 58.0 (56.1, 60.0) | 56.9 (56.1, 57.6) | 64.0 (62.0, 66.1) | <0.001*** |
| Race, *n* (%) |  |  |  | <0.001*** |
| Mexican American | 12.64 (11.25, 14.03) | 13.13 (11.63, 14.62) | 10.21 (8.63, 11.79) |  |
| Non-Hispanic White | 58.75 (55.13, 62.37) | 58.89 (56.65, 61.13) | 58.05 (54.71, 61.39) |  |
| Non-Hispanic Black | 12.98 (11.86, 14.10) | 12.15 (10.96, 13.34) | 17.13 (15.18, 19.08) |  |
| Other Hispanic | 7.53 (6.39, 8.66) | 7.61 (6.48, 8.74) | 7.10 (5.80, 8.41) |  |
| Other | 8.10 (7.39, 8.82) | 8.22 (7.47, 8.97) | 7.51 (6.40, 8.61) |  |
| Smoking, *n* (%) | 25.69 (24.41, 26.97) | 24.79 (23.66, 25.91) | 30.23 (28.10, 32.36) | <0.001*** |
| Alcohol users, *n* (%) | 78.26 (75.60, 80.92) | 78.45 (77.21, 79.70) | 77.28 (75.30, 79.26) | 0.21 |
| Education level, *n* (%) |  |  |  | <0.001*** |
| Below high school | 5.74 (5.23, 6.25) | 5.55 (5.03, 6.08) | 6.68 (5.77, 7.59) |  |
| High school | 38.67 (36.81, 40.53) | 38.06 (36.61, 39.52) | 41.72 (39.60, 43.83) |  |
| Above high school | 55.59 (53.30, 57.88) | 56.38 (54.74, 58.03) | 51.60 (49.36, 53.84) |  |
| SBP, mmHg | 118.12 (117.79, 118.45) | 114.37 (114.11, 114.63) | 137.01 (136.20, 137.82) | <0.001*** |
| DBP, mmHg | 70.89 (70.58, 71.21) | 68.97 (68.68, 69.26) | 80.59 (79.94, 81.23) | <0.001*** |
| Diabetes, *n* (%) | 3.28 (2.96, 3.60) | 2.41 (2.13, 2.69) | 7.66 (6.54, 8.78) | <0.001*** |
| FBG, mmol/L | 5.55 (5.53, 5.57) | 5.48 (5.46, 5.50) | 5.89 (5.81, 5.98) | <0.001*** |
| HbA1c, % | 5.36 (5.35, 5.38) | 5.32 (5.31, 5.33) | 5.56 (5.52, 5.61) | <0.001*** |
| eGFR, ml/min/1.73m^2^ | 104.05 (103.60, 104.50) | 105.03 (104.54, 105.52) | 99.11 (98.41, 99.81) | 0.04* |
| TG, mmol/L | 2.08 (2.04, 2.11) | 1.99 (1.95, 2.04) | 2.49 (2.39, 2.59) | <0.001*** |
| TC, mmol/L | 5.00 (4.98, 5.02) | 4.93 (4.90, 4.95) | 5.36 (5.31, 5.42) | <0.001*** |
| LDL-C, mmol/L | 2.72 (2.70, 2.74) | 2.68 (2.66, 2.70) | 2.93 (2.88, 2.99) | <0.001*** |
| HDL-C, mmol/L | 1.34 (1.33, 1.35) | 1.35 (1.34, 1.36) | 1.31 (1.30, 1.33) | <0.001*** |
| RBC, ×10^9^/L | 4.83 (4.81, 4.84) | 4.81 (4.80, 4.82) | 4.90 (4.88, 4.92) | <0.001*** |
| WBC, ×10^9^/L | 7.19 (7.15, 7.24) | 7.15 (7.10, 7.19) | 7.43 (7.33, 7.53) | <0.001*** |
| NE, ×10^9^/L | 4.23 (4.19, 4.26) | 4.19 (4.16, 4.23) | 4.40 (4.32, 4.47) | <0.001*** |
| Monocyte, ×10^9^/L | 0.55 (0.55, 0.56) | 0.55 (0.55, 0.55) | 0.57 (0.56, 0.58) | <0.001*** |
| LY, ×10^9^/L | 2.17 (2.16, 2.19) | 2.16 (2.15, 2.18) | 2.20 (2.17, 2.23) | 0.03* |
| PLT, ×10^6^/L | 255.39 (253.89, 256.88) | 254.63 (253.10, 256.17) | 259.17 (256.23, 262.10) | 0.003** |
| Hemoglobin, g/L | 14.58 (14.53, 14.62) | 14.53 (14.49, 14.58) | 14.79 (14.72, 14.86) | <0.001*** |
| HEI-2015 | 48.99 (48.59, 49.40) | 49.07 (48.65, 49.50) | 48.60 (47.97, 49.22) | 0.14 |

Continuous variables are presented as the mean and 95% confidence interval, category variables are presented as the proportion and 95% confidence interval. SBP, systolic blood pressure; DBP, diastolic blood pressure; FBG, fasting blood glucose; HbA1c, glycated hemoglobin; eGFR, estimated glomerular filtration rate; BMI, body mass index; WC, waist circumference; TG, triglycerides; TC, total cholesterol; LDL-C, low-density lipoprotein cholesterol; HDL-C, high-density lipoprotein cholesterol; RBC, red blood cells; WBC, white blood cells; NE, neutrophils; LY, lymphocytes; PLT, platelets. Ethnic information is presented in alphabetical order. *** *P* value<0.001.
